# Surgical anatomy of microsurgical 3-level anterior cervical discectomy and fusion C4–C7

**DOI:** 10.17305/bjbms.2020.4895

**Published:** 2021-06

**Authors:** Domagoj Gajski, Alicia R. Dennis, Kenan I. Arnautović

**Affiliations:** 1 Department of Neurosurgery, University Hospital Center Sestre Milosrdnice, Zagreb, Croatia; 2 Department of Anatomy and Physiology, University of Applied Health Sciences, Zagreb, Croatia; 3 School of Dental Medicine, University of Zagreb, Zagreb, Croatia; 4 Semmes Murphey Neurologic and Spine Institute, Memphis, Tennessee, United States; 5 Department of Neurosurgery, University of Tennessee Health Science Center, Memphis, Tennessee, United States

**Keywords:** Anterior cervical spine, ACDF, discectomy, fusion, allograft

## Abstract

Anterior cervical discectomy and fusion (ACDF) is one of the most common spinal procedures, frequently used for the treatment of cervical spine degenerative diseases. It was first described in 1958. Interestingly, to our knowledge, 3-level ACDF has not been previously published as a peer-reviewed video case with a detailed description of intraoperative microsurgical anatomy. In this video, we present the case of a 33-year-old male who presented with a combination of myelopathy (hyperreflexia and long tract signs in the upper and lower extremities) and bilateral radiculopathy of the upper extremities. He had been previously treated conservatively with physical therapy and pain management for 6 months without success. We performed 3-level microsurgical ACDF from C4 to C7. All 3 levels were decompressed, and bone allografts were placed to achieve intervertebral body fusion. A titanium plate was utilized from C4 to C7 for internal fixation. The patient was discharged home on the first postoperative day. His pain, numbness and tingling resolved, as well as his myelopathy. No perioperative complications were encountered. Herein we present the surgical anatomy of our operative technique including certain technical tips. Written consent was obtained directly from the patient.

## VIDEO ARTICLE LINK

https://www.bjbms.org/ojs/index.php/bjbms/article/view/4895

Video 1

## VIDEO ANNOTATIONS

01:13—opening the surgery site

02:29—positioning of retractors

03:02—start of 3-level discectomy

06:04—allograft placement and fixation

08:20—drain placement and closure

## INTRODUCTION

Anterior cervical discectomy and fusion (ACDF) is one of the most common spinal procedures, frequently used for the treatment of cervical spine degenerative diseases. It was first described in 1958 [1,2]. Interestingly, to our knowledge, 3-level ACDF has not been previously published as a peer-reviewed video case with detailed description of intraoperative microsurgical anatomy [3-7].

## VIDEO TRANSCRIPT

This is a video of a micro surgical 3-level anterior cervical discectomy and fusion from C4 to C7. This patient is a 34-year-old African-American man with severe bilateral radiculopathy in C6 and C7 distribution and bilateral upper and lower extremity hyperreflexia with numbness and tingling of the arms and legs for three years. His magnetic resonance imaging (MRI) showed multiple level cervical spinal spondylosis with the most pronounced levels at C4-5, C5-6, and C6-7, with severe bilateral neural foraminal narrowing at all three levels. Based on his MRI, the flexion-extension X-rays of cervical spine, as well as clinical symptoms on the neurological exam, we discussed options of treatment with the patient. He has already failed physical therapy for three months as well as two epidural nerve blocks. He wished to proceed with surgery flexion and extension.

C spine X-rays showed significant anterior osteophytes at C5-6 and C6-7, as well as posterior osteophytes at 4-5 and 5-6 without spinal alignment disturbance. Patient’s operative position with supine. We placed a chin strap with traction of seven pounds of weight. We identified the midline and then medial border of the left-sided sternocleidomastoid muscle. Skin incision was approximately two inches long. His left lateral anterior neck was sterilely prepped and draped.

After skin incision, we incised the platysma muscle. We identified the left-sided omohyoid muscle and dissected it. Stitches were placed proximally and distally. Then we incised the muscle and divided it with a Bovie cautery. The edges of the muscle were retracted superiorly and inferiorly serving as a form of superior-inferior retraction. We continued dissection between the neck organs medially and neurovascular bundle laterally. The carotid artery and jugular vein were clearly visible on the left side.

After reaching the anterior surface of the cervical spine, the deep cervical fascia was opened and the left and right anterior longus colli muscles were identified. Bovie cautery at the power of 15 was used to incise at the medial aspect of the left and right anterior longus colli muscles. A bent needle was used to identify the highest level planned for surgery at C4-5, which is demonstrated on the intraoperative lateral C spine X-ray.

Following this, the latero-lateral retractor was placed in position at C5-6 at the center of the exposure and then the Kerlix was placed on the right retractor blade. An additional two pounds of weight was placed on it to prevent the right-sided retractor from sliding. Superior and inferior retraction were obtained with a pair of Trimline retractors. Final aspect of exposure is seen with the surgical field prepared for a three level anterior microsurgical discectomy with anterior aspect of the vertebral bodies from C4 to C7 with adjacent intervertebral spaces.

We started the anterior cervical discectomy at the level of C6-7. We then used the Midas Rex drill with the M8 tip and shaved off cartilaginous endplates from the upper and lower surface of the vertebral bodies. Once the bone bleeding at the junction of the cartilaginous endplates and the vertebral body was encountered, we stopped the drilling. We then used one millimeter and two millimeter Kerrison Rongeurs to take down the posterior longitudinal ligament and any adjacent disc osteophytes on both sides. We decompressed both nerve roots to the right and the left. Gelfoam powder was used for hemostasis continuously. We simultaneously prepared and flattened the anterior surface of the vertebral bodies to be able to appropriately accommodate the plate.

The next level was at C5-6. Again we used the Midas Rex drill with an M8 tip to shave off the inferior lip of the C5 vertebral body. We then used the number 15 blade to incise the anterior longitudinal ligament and emptied the soft disc material with pituitary rongeurs. We exposed the medial aspect of the bilateral uncinate processes, and then again using the Midas Rex drill with the M8 tip to shave off cartilaginous endplates of C5 and C6 vertebral bodies and then down to the disc osteophytes inferiorly.

We went drilling all the way to medial aspects of both uncinated processes inferiorly. We used one and two millimeter Kerrison Rongeurs to decompress the dura, including the bilateral nerve roots at C5-6. We confirmed the decompression with blunt micro nerve hooks. We made sure to undercut superiorly and inferiorly the intervertebral disk space and osteophytes providing adequate decompression. The same was repeated at the C4 and the C5 levels. We drilled out the inferior lip of the superior vertebral body and then we resected the soft material with pituitary rongeurs.

We again identified the medial aspect of the uncinated processes both on the right and on the left side. Again the drilling of the cartilaginous endplates was done and then Kerrison Rongeurs of one and two millimeters completed the resection of the posterior longitudinal ligament and the disc osteophytes. Again at this level, good decompression was achieved, removing the soft disc material and the disc osteophytes. Note the rectangular shape of intervertebral disk space is prepared for allografts and the flattened interior surfaces of the vertebral bodies. This is a final view after microsurgical discectomy at the C4-7 levels.

We then started using graft-size trials starting from C6 to C7. Appropriate size of cortical allografts filled up with bone putty were placed in position, achieving intervertebral body fusion at C6-7, and they were tapped down just a millimeter below the anterior surface of the anterior cervical spine surface. We continued using gel foam powder for hemostasis. The same was repeated at the C5-6 level.

A trial was used to determine the size of the allograft and again allograft was placed in position, achieving intervertebral body fusion at the C5-6 level. Finally, the same was done at the C4-5 level and this is a final view of intervertebral body fusion at C4-7.

At this point, weights from cervical traction were taken off. The grafts were tightly fitting and placed in position at each level. We then placed the plate in position, making sure that the plate is fitting well and stable along the anterior surface of the cervical spine from C4 to C7. The plate was positioned a few millimeters below the superior endplate of C4, as well as above the inferior endplate of C7. To make sure that the plate is in the midline, we watched the distance between uncinate processes and plate at all three levels. The plate was secured to the vertebral body of C6 with pins. The drill was used to create pathway for screws and variable titanium screws were placed in position first at C4. Appropriate size of screws was chosen measuring it relatively to the size of the drill, which is 13 millimeters. This can be visualized on the lateral X-rays.

We placed the C4 screws in position. We placed the screws into the vertebral body of C7 and then took out the safety pins. We repeated this process at C5 and C6 and confirmed with lateral X-rays. Once the screws were nice and tight and in position, they were locked. Irrigation with copious amounts of antibiotic saline was done. Any bleeding was easily controlled by bipolar coagulation and Gelfoam powder.

We then inspected the jugular vein and the internal carotid artery on the left side as well as medial wall of the esophagus. We then placed the drain in position, externalizing it via a separate outlet and securing with a 2.0 silk stitch. The omohyoid muscle was returned to position, suturing it together, and then the wound was closed with subcutaneous 2-0 Vicryl and 3-0 Vicryl intermittent stitches. Steri-strips, telfa, and Tegaderm were used for dressing.

Final AP and lateral X-rays confirmed good positioning of the plate screws and allografts. Patient was discharged home on the first postoperative day and he returned for a wound check and C spine X-rays two weeks after surgery. He was kept in a soft collar for two weeks and then the collar was weaned off in the following week. His numbness and tingling resolved immediately after surgery and his hyperreflexia resolved as well.
